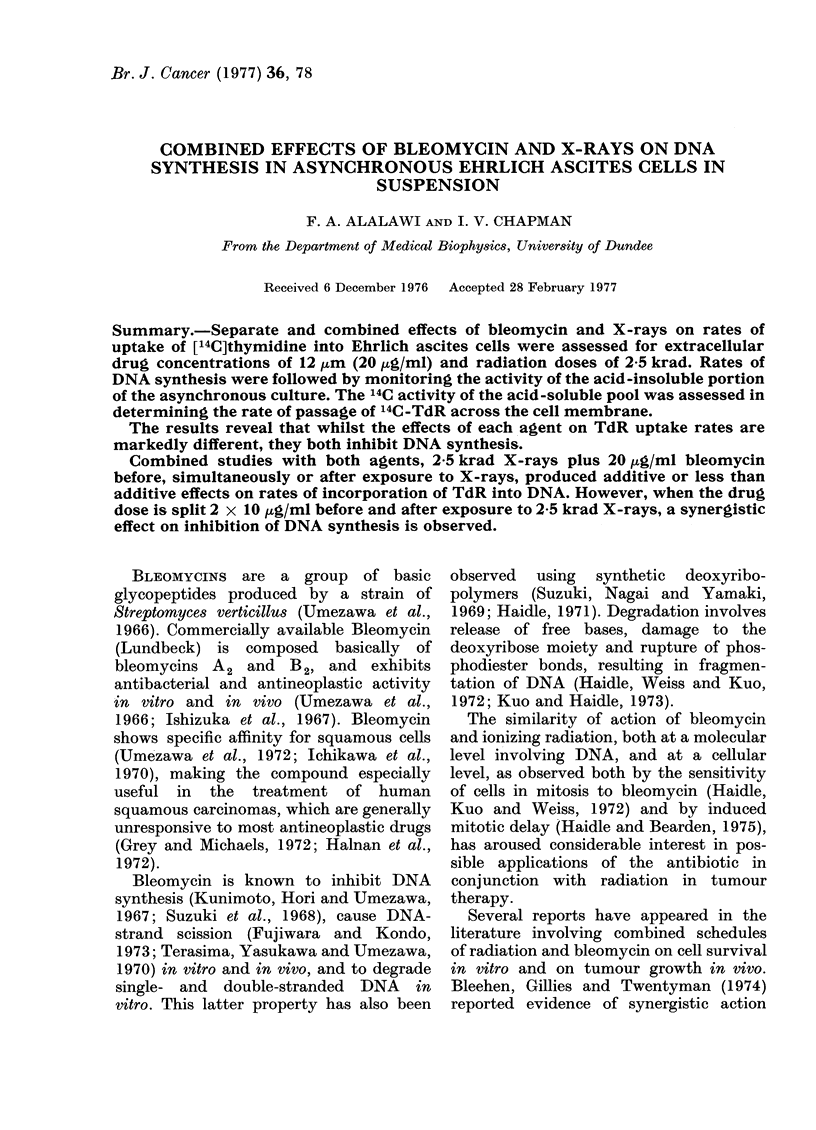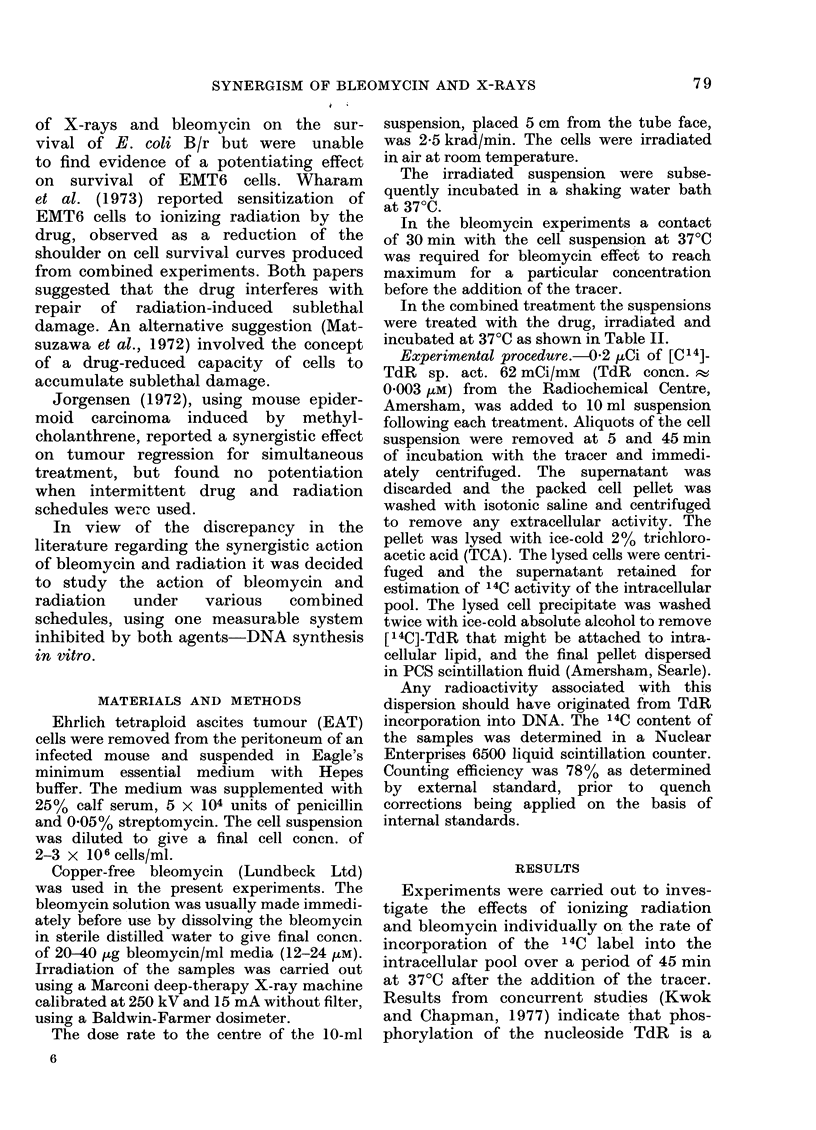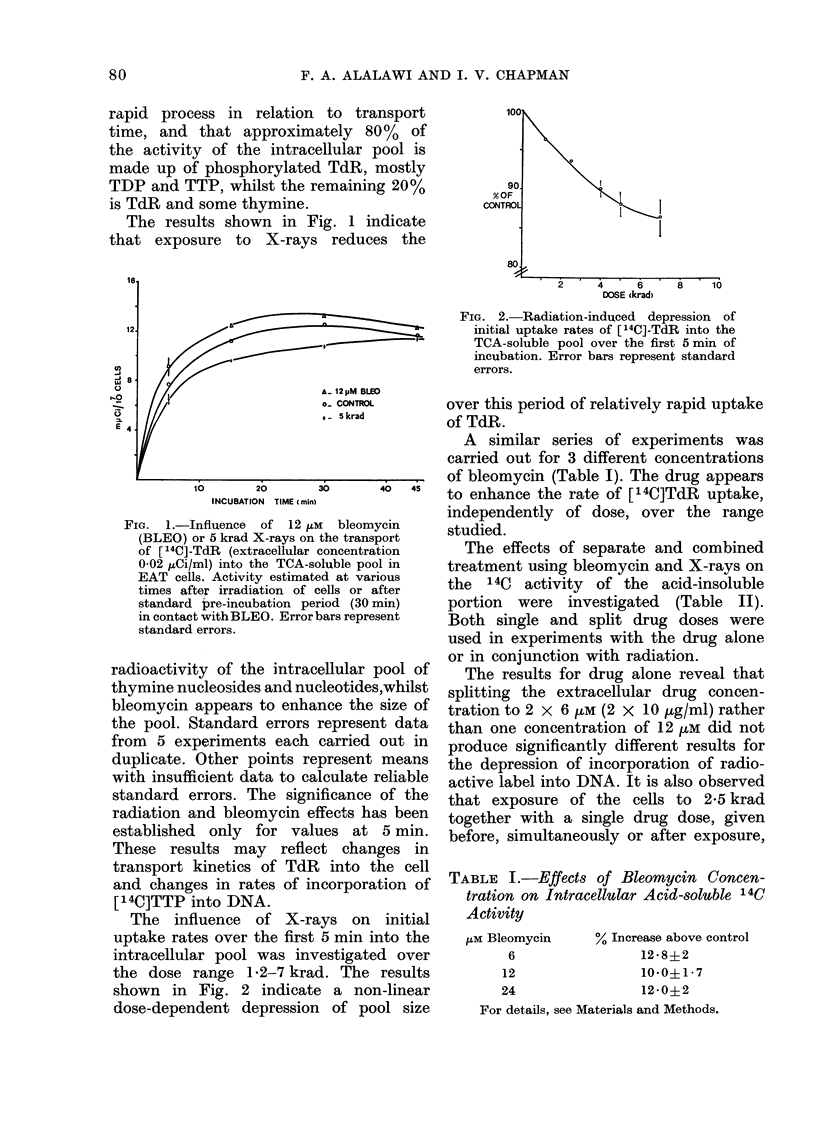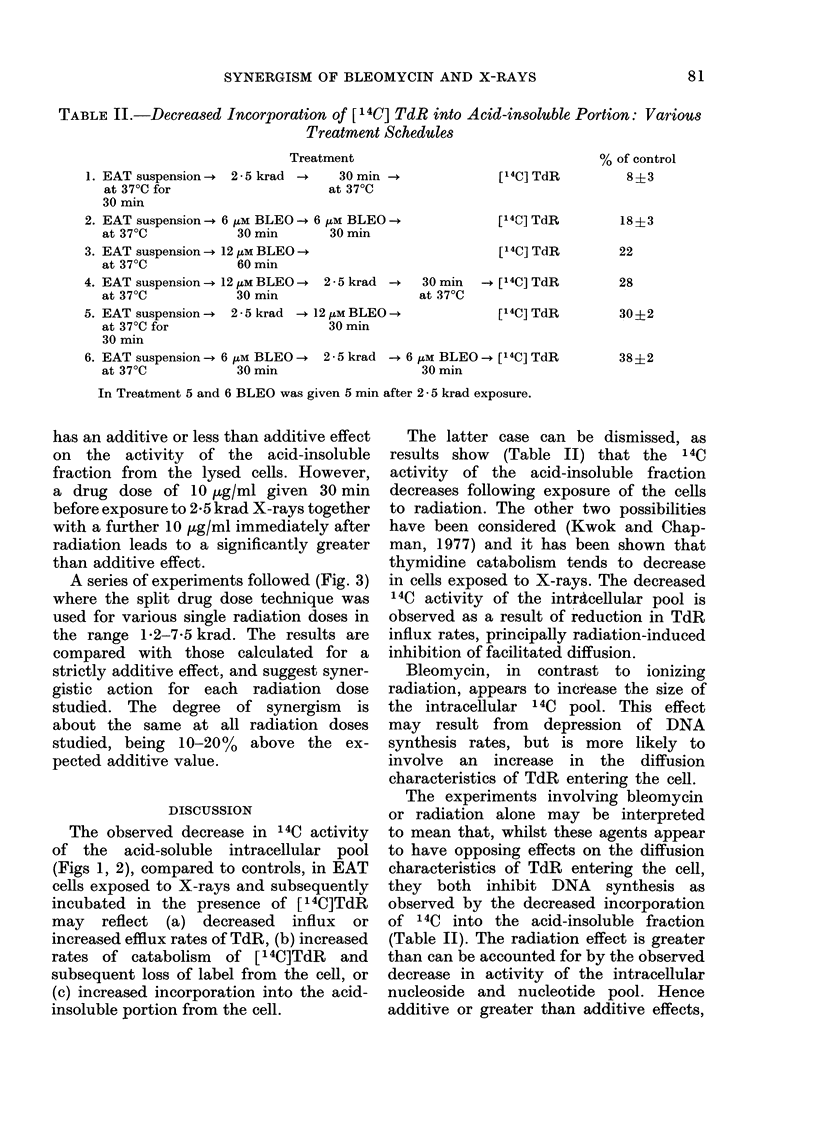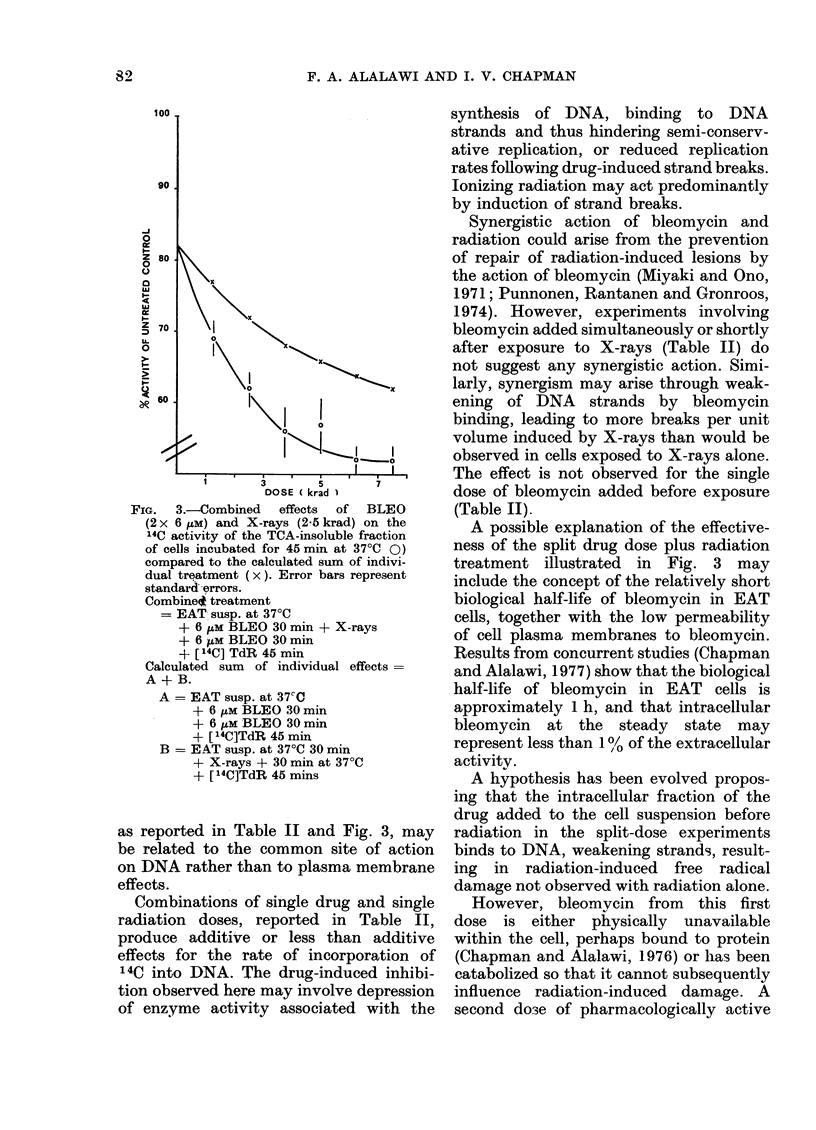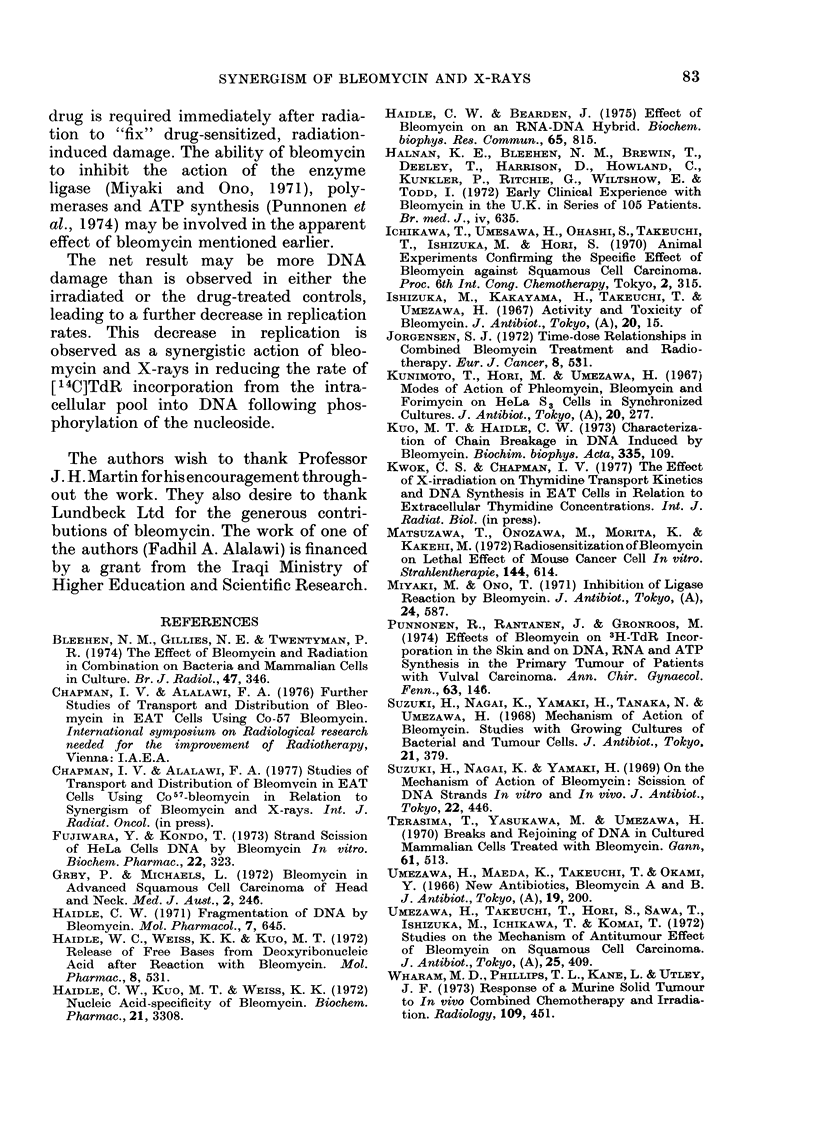# Combined effects of bleomycin and X-rays on DNA synthesis in asynchronous Ehrlich ascites cells in suspension.

**DOI:** 10.1038/bjc.1977.157

**Published:** 1977-07

**Authors:** F. A. Alalawi, I. V. Chapman

## Abstract

Separate and combined effects of bleomycin and X-rays on rates of uptake of [14C]thymidine into Ehrlich ascites cells were assessed for extracellular drug concentrations of 12 micron (20 microgram/ml) and radiation doses of 2-5 krad. Rates of DNA synthesis were followed by monitoring the activity of the acid-insoluble portion of the asynchronous culture. The 14C activity of the acid-soluble pool was assessed in determining the rate of passage of 14C-TdR across the cell membrane. The results reveal that whilst the effects of each agent on TdR uptake rates are markedly different, they both inhibit DNA synthesis. Combined studies with both agents, 2-5 krad X-rays plus 20 microgram/ml bleomycin before, simultaneously or after exposure to X-rays, produced additive or less than additive effects on rates of incorporation of TdR into DNA. However, when the drug dose is split 2 X 10 microgram/ml before and after exposure to 2-5 krad X-rays, a synergistic effect on inhibition of DNA synthesis is observed.


*Br. J. Cancer* (1977) **36**, 78

### COMBINED EFFECTS OF BLEOMYCIN AND X-RAYS ON DNA SYNTHESIS IN ASYNCHRONOUS EHRLICH ASCITES CELLS IN SUSPENSION

F. A. ALALAWI AND I. V. CHAPMAN

*From the Department of Medical Biophysics, University of Dundee*

Received 6 December 1976 Accepted 28 February 1977

**Summary.—Separate and combined effects of bleomycin and X-rays on rates of uptake of [$^{14}$C]thymidine into Ehrlich ascites cells were assessed for extracellular drug concentrations of 12 $\mu$m (20 $\mu$g/ml) and radiation doses of 2·5 krad. Rates of DNA synthesis were followed by monitoring the activity of the acid-insoluble portion of the asynchronous culture. The $^{14}$C activity of the acid-soluble pool was assessed in determining the rate of passage of $^{14}$C-TdR across the cell membrane.**

**The results reveal that whilst the effects of each agent on TdR uptake rates are markedly different, they both inhibit DNA synthesis.**

**Combined studies with both agents, 2·5 krad X-rays plus 20 $\mu$g/ml bleomycin before, simultaneously or after exposure to X-rays, produced additive or less than additive effects on rates of incorporation of TdR into DNA. However, when the drug dose is split 2 $\times$ 10 $\mu$g/ml before and after exposure to 2·5 krad X-rays, a synergistic effect on inhibition of DNA synthesis is observed.**

BLEOMYCINS are a group of basic glycopeptides produced by a strain of *Streptomyces verticillus* (Umezawa *et al.*, 1966). Commercially available Bleomycin (Lundbeck) is composed basically of bleomycins $A_2$ and $B_2$, and exhibits antibacterial and antineoplastic activity *in vitro* and *in vivo* (Umezawa *et al.*, 1966; Ishizuka *et al.*, 1967). Bleomycin shows specific affinity for squamous cells (Umezawa *et al.*, 1972; Ichikawa *et al.*, 1970), making the compound especially useful in the treatment of human squamous carcinomas, which are generally unresponsive to most antineoplastic drugs (Grey and Michaels, 1972; Halnan *et al.*, 1972).

Bleomycin is known to inhibit DNA synthesis (Kunimoto, Hori and Umezawa, 1967; Suzuki *et al.*, 1968), cause DNA-strand scission (Fujiwara and Kondo, 1973; Terasima, Yasukawa and Umezawa, 1970) *in vitro* and *in vivo*, and to degrade single- and double-stranded DNA *in vitro*. This latter property has also been observed using synthetic deoxyribopolymers (Suzuki, Nagai and Yamaki, 1969; Haidle, 1971). Degradation involves release of free bases, damage to the deoxyribose moiety and rupture of phosphodiester bonds, resulting in fragmentation of DNA (Haidle, Weiss and Kuo, 1972; Kuo and Haidle, 1973).

The similarity of action of bleomycin and ionizing radiation, both at a molecular level involving DNA, and at a cellular level, as observed both by the sensitivity of cells in mitosis to bleomycin (Haidle, Kuo and Weiss, 1972) and by induced mitotic delay (Haidle and Bearden, 1975), has aroused considerable interest in possible applications of the antibiotic in conjunction with radiation in tumour therapy.

Several reports have appeared in the literature involving combined schedules of radiation and bleomycin on cell survival *in vitro* and on tumour growth *in vivo*. Bleehen, Gillies and Twentyman (1974) reported evidence of synergistic action of X-rays and bleomycin on the survival of *E. coli* B/r but were unable to find evidence of a potentiating effect on survival of EMT6 cells. Wharam *et al.* (1973) reported sensitization of EMT6 cells to ionizing radiation by the drug, observed as a reduction of the shoulder on cell survival curves produced from combined experiments. Both papers suggested that the drug interferes with repair of radiation-induced sublethal damage. An alternative suggestion (Matsuzawa *et al.*, 1972) involved the concept of a drug-reduced capacity of cells to accumulate sublethal damage.

Jorgensen (1972), using mouse epidermoid carcinoma induced by methylcholanthrene, reported a synergistic effect on tumour regression for simultaneous treatment, but found no potentiation when intermittent drug and radiation schedules were used.

In view of the discrepancy in the literature regarding the synergistic action of bleomycin and radiation it was decided to study the action of bleomycin and radiation under various combined schedules, using one measurable system inhibited by both agents—DNA synthesis *in vitro*.

### MATERIALS AND METHODS

Ehrlich tetraploid ascites tumour (EAT) cells were removed from the peritoneum of an infected mouse and suspended in Eagle's minimum essential medium with Hepes buffer. The medium was supplemented with 25% calf serum, $5 \times 10^4$ units of penicillin and 0·05% streptomycin. The cell suspension was diluted to give a final cell concn. of $2–3 \times 10^6$ cells/ml.

Copper-free bleomycin (Lundbeck Ltd) was used in the present experiments. The bleomycin solution was usually made immediately before use by dissolving the bleomycin in sterile distilled water to give final concn. of 20–40 μg bleomycin/ml media (12–24 μM). Irradiation of the samples was carried out using a Marconi deep-therapy X-ray machine calibrated at 250 kV and 15 mA without filter, using a Baldwin-Farmer dosimeter.

The dose rate to the centre of the 10-ml suspension, placed 5 cm from the tube face, was 2·5 krad/min. The cells were irradiated in air at room temperature.

The irradiated suspension were subsequently incubated in a shaking water bath at 37°C.

In the bleomycin experiments a contact of 30 min with the cell suspension at 37°C was required for bleomycin effect to reach maximum for a particular concentration before the addition of the tracer.

In the combined treatment the suspensions were treated with the drug, irradiated and incubated at 37°C as shown in Table II.

*Experimental procedure.*—0·2 μCi of [$C^{14}$]-TdR sp. act. 62 mCi/mM (TdR concn. ≈ 0·003 μM) from the Radiochemical Centre, Amersham, was added to 10 ml suspension following each treatment. Aliquots of the cell suspension were removed at 5 and 45 min of incubation with the tracer and immediately centrifuged. The supernatant was discarded and the packed cell pellet was washed with isotonic saline and centrifuged to remove any extracellular activity. The pellet was lysed with ice-cold 2% trichloroacetic acid (TCA). The lysed cells were centrifuged and the supernatant retained for estimation of $^{14}C$ activity of the intracellular pool. The lysed cell precipitate was washed twice with ice-cold absolute alcohol to remove [$^{14}C$]-TdR that might be attached to intracellular lipid, and the final pellet dispersed in PCS scintillation fluid (Amersham, Searle).

Any radioactivity associated with this dispersion should have originated from TdR incorporation into DNA. The $^{14}C$ content of the samples was determined in a Nuclear Enterprises 6500 liquid scintillation counter. Counting efficiency was 78% as determined by external standard, prior to quench corrections being applied on the basis of internal standards.

### RESULTS

Experiments were carried out to investigate the effects of ionizing radiation and bleomycin individually on the rate of incorporation of the $^{14}C$ label into the intracellular pool over a period of 45 min at 37°C after the addition of the tracer. Results from concurrent studies (Kwok and Chapman, 1977) indicate that phosphorylation of the nucleoside TdR is a rapid process in relation to transport time, and that approximately 80% of the activity of the intracellular pool is made up of phosphorylated TdR, mostly TDP and TTP, whilst the remaining 20% is TdR and some thymine.

The results shown in Fig. 1 indicate that exposure to X-rays reduces the

FIG. 1.—Influence of 12 $\mu$M bleomycin (BLEO) or 5 krad X-rays on the transport of [$^{14}$C]-TdR (extracellular concentration 0·02 $\mu$Ci/ml) into the TCA-soluble pool in EAT cells. Activity estimated at various times after irradiation of cells or after standard pre-incubation period (30 min) in contact with BLEO. Error bars represent standard errors.

radioactivity of the intracellular pool of thymine nucleosides and nucleotides, whilst bleomycin appears to enhance the size of the pool. Standard errors represent data from 5 experiments each carried out in duplicate. Other points represent means with insufficient data to calculate reliable standard errors. The significance of the radiation and bleomycin effects has been established only for values at 5 min. These results may reflect changes in transport kinetics of TdR into the cell and changes in rates of incorporation of [$^{14}$C]TTP into DNA.

The influence of X-rays on initial uptake rates over the first 5 min into the intracellular pool was investigated over the dose range 1·2–7 krad. The results shown in Fig. 2 indicate a non-linear dose-dependent depression of pool size

FIG. 2.—Radiation-induced depression of initial uptake rates of [$^{14}$C]-TdR into the TCA-soluble pool over the first 5 min of incubation. Error bars represent standard errors.

over this period of relatively rapid uptake of TdR.

A similar series of experiments was carried out for 3 different concentrations of bleomycin (Table I). The drug appears to enhance the rate of [$^{14}$C]TdR uptake, independently of dose, over the range studied.

The effects of separate and combined treatment using bleomycin and X-rays on the $^{14}$C activity of the acid-insoluble portion were investigated (Table II). Both single and split drug doses were used in experiments with the drug alone or in conjunction with radiation.

The results for drug alone reveal that splitting the extracellular drug concentration to 2 × 6 $\mu$M (2 × 10 $\mu$g/ml) rather than one concentration of 12 $\mu$M did not produce significantly different results for the depression of incorporation of radioactive label into DNA. It is also observed that exposure of the cells to 2·5 krad together with a single drug dose, given before, simultaneously or after exposure,

TABLE I.—*Effects of Bleomycin Concentration on Intracellular Acid-soluble $^{14}$C Activity*

| $\mu$M Bleomycin | % Increase above control |
|---|---|
| 6 | 12·8±2 |
| 12 | 10·0±1·7 |
| 24 | 12·0±2 |

For details, see Materials and Methods.

TABLE II.—*Decreased Incorporation of [$^{14}$C] TdR into Acid-insoluble Portion: Various Treatment Schedules*

| | Treatment | | | | | % of control |
|---|---|---|---|---|---|---|
| 1. | EAT suspension → at 37°C for 30 min | 2·5 krad → | 30 min → at 37°C | | [$^{14}$C] TdR | 8±3 |
| 2. | EAT suspension → at 37°C | 6 μM BLEO → 30 min | 6 μM BLEO → 30 min | | [$^{14}$C] TdR | 18±3 |
| 3. | EAT suspension → at 37°C | 12 μM BLEO → 60 min | | | [$^{14}$C] TdR | 22 |
| 4. | EAT suspension → at 37°C | 12 μM BLEO → 30 min | 2·5 krad → | 30 min → at 37°C | [$^{14}$C] TdR | 28 |
| 5. | EAT suspension → at 37°C for 30 min | 2·5 krad → | 12 μM BLEO → 30 min | | [$^{14}$C] TdR | 30±2 |
| 6. | EAT suspension → at 37°C | 6 μM BLEO → 30 min | 2·5 krad → | 6 μM BLEO → 30 min | [$^{14}$C] TdR | 38±2 |

In Treatment 5 and 6 BLEO was given 5 min after 2·5 krad exposure.

has an additive or less than additive effect on the activity of the acid-insoluble fraction from the lysed cells. However, a drug dose of 10 μg/ml given 30 min before exposure to 2·5 krad X-rays together with a further 10 μg/ml immediately after radiation leads to a significantly greater than additive effect.

A series of experiments followed (Fig. 3) where the split drug dose technique was used for various single radiation doses in the range 1·2–7·5 krad. The results are compared with those calculated for a strictly additive effect, and suggest synergistic action for each radiation dose studied. The degree of synergism is about the same at all radiation doses studied, being 10–20% above the expected additive value.

### DISCUSSION

The observed decrease in $^{14}$C activity of the acid-soluble intracellular pool (Figs 1, 2), compared to controls, in EAT cells exposed to X-rays and subsequently incubated in the presence of [$^{14}$C]TdR may reflect (a) decreased influx or increased efflux rates of TdR, (b) increased rates of catabolism of [$^{14}$C]TdR and subsequent loss of label from the cell, or (c) increased incorporation into the acid-insoluble portion from the cell.

The latter case can be dismissed, as results show (Table II) that the $^{14}$C activity of the acid-insoluble fraction decreases following exposure of the cells to radiation. The other two possibilities have been considered (Kwok and Chapman, 1977) and it has been shown that thymidine catabolism tends to decrease in cells exposed to X-rays. The decreased $^{14}$C activity of the intracellular pool is observed as a result of reduction in TdR influx rates, principally radiation-induced inhibition of facilitated diffusion.

Bleomycin, in contrast to ionizing radiation, appears to increase the size of the intracellular $^{14}$C pool. This effect may result from depression of DNA synthesis rates, but is more likely to involve an increase in the diffusion characteristics of TdR entering the cell.

The experiments involving bleomycin or radiation alone may be interpreted to mean that, whilst these agents appear to have opposing effects on the diffusion characteristics of TdR entering the cell, they both inhibit DNA synthesis as observed by the decreased incorporation of $^{14}$C into the acid-insoluble fraction (Table II). The radiation effect is greater than can be accounted for by the observed decrease in activity of the intracellular nucleoside and nucleotide pool. Hence additive or greater than additive effects,

FIG. 3.—Combined effects of BLEO (2 × 6 μM) and X-rays (2·5 krad) on the $^{14}C$ activity of the TCA-insoluble fraction of cells incubated for 45 min at 37°C ○) compared to the calculated sum of individual treatment (×). Error bars represent standard errors.
Combined treatment
= EAT susp. at 37°C
+ 6 μM BLEO 30 min + X-rays
+ 6 μM BLEO 30 min
+ [$^{14}$C] TdR 45 min
Calculated sum of individual effects = A + B.
A = EAT susp. at 37°C
+ 6 μM BLEO 30 min
+ 6 μM BLEO 30 min
+ [$^{14}$C]TdR 45 min
B = EAT susp. at 37°C 30 min
+ X-rays + 30 min at 37°C
+ [$^{14}$C]TdR 45 mins as reported in Table II and Fig. 3, may be related to the common site of action on DNA rather than to plasma membrane effects.

Combinations of single drug and single radiation doses, reported in Table II, produce additive or less than additive effects for the rate of incorporation of $^{14}C$ into DNA. The drug-induced inhibition observed here may involve depression of enzyme activity associated with the synthesis of DNA, binding to DNA strands and thus hindering semi-conservative replication, or reduced replication rates following drug-induced strand breaks. Ionizing radiation may act predominantly by induction of strand breaks.

Synergistic action of bleomycin and radiation could arise from the prevention of repair of radiation-induced lesions by the action of bleomycin (Miyaki and Ono, 1971; Punnonen, Rantanen and Gronroos, 1974). However, experiments involving bleomycin added simultaneously or shortly after exposure to X-rays (Table II) do not suggest any synergistic action. Similarly, synergism may arise through weakening of DNA strands by bleomycin binding, leading to more breaks per unit volume induced by X-rays than would be observed in cells exposed to X-rays alone. The effect is not observed for the single dose of bleomycin added before exposure (Table II).

A possible explanation of the effectiveness of the split drug dose plus radiation treatment illustrated in Fig. 3 may include the concept of the relatively short biological half-life of bleomycin in EAT cells, together with the low permeability of cell plasma membranes to bleomycin. Results from concurrent studies (Chapman and Alalawi, 1977) show that the biological half-life of bleomycin in EAT cells is approximately 1 h, and that intracellular bleomycin at the steady state may represent less than 1% of the extracellular activity.

A hypothesis has been evolved proposing that the intracellular fraction of the drug added to the cell suspension before radiation in the split-dose experiments binds to DNA, weakening strands, resulting in radiation-induced free radical damage not observed with radiation alone. However, bleomycin from this first dose is either physically unavailable within the cell, perhaps bound to protein (Chapman and Alalawi, 1976) or has been catabolized so that it cannot subsequently influence radiation-induced damage. A second dose of pharmacologically active drug is required immediately after radiation to "fix" drug-sensitized, radiation-induced damage. The ability of bleomycin to inhibit the action of the enzyme ligase (Miyaki and Ono, 1971), polymerases and ATP synthesis (Punnonen *et al.*, 1974) may be involved in the apparent effect of bleomycin mentioned earlier.

The net result may be more DNA damage than is observed in either the irradiated or the drug-treated controls, leading to a further decrease in replication rates. This decrease in replication is observed as a synergistic action of bleomycin and X-rays in reducing the rate of [$^{14}$C]TdR incorporation from the intracellular pool into DNA following phosphorylation of the nucleoside.

The authors wish to thank Professor J. H. Martin for his encouragement throughout the work. They also desire to thank Lundbeck Ltd for the generous contributions of bleomycin. The work of one of the authors (Fadhil A. Alalawi) is financed by a grant from the Iraqi Ministry of Higher Education and Scientific Research.

### REFERENCES

Bleehen, N. M., Gillies, N. E. & Twentyman, P. R. (1974) The Effect of Bleomycin and Radiation in Combination on Bacteria and Mammalian Cells in Culture. *Br. J. Radiol.*, **47**, 346.

Chapman, I. V. & Alalawi, F. A. (1976) Further Studies of Transport and Distribution of Bleomycin in EAT Cells Using Co-57 Bleomycin. *International symposium on Radiological research needed for the improvement of Radiotherapy*, Vienna: I.A.E.A.

Chapman, I. V. & Alalawi, F. A. (1977) Studies of Transport and Distribution of Bleomycin in EAT Cells Using $Co^{57}$-bleomycin in Relation to Synergism of Bleomycin and X-rays. *Int. J. Radiat. Oncol.* (in press).

Fujiwara, Y. & Kondo, T. (1973) Strand Scission of HeLa Cells DNA by Bleomycin *In vitro*. *Biochem. Pharmac.*, **22**, 323.

Greby, P. & Michaels, L. (1972) Bleomycin in Advanced Squamous Cell Carcinoma of Head and Neck. *Med. J. Aust.*, **2**, 246.

Haidle, C. W. (1971) Fragmentation of DNA by Bleomycin. *Mol. Pharmacol.*, **7**, 645.

Haidle, W. C., Weiss, K. K. & Kuo, M. T. (1972) Release of Free Bases from Deoxyribonucleic Acid after Reaction with Bleomycin. *Mol. Pharmac.*, **8**, 531.

Haidle, C. W., Kuo, M. T. & Weiss, K. K. (1972) Nucleic Acid-specificity of Bleomycin. *Biochem. Pharmac.*, **21**, 3308.

Haidle, C. W. & Bearden, J. (1975) Effect of Bleomycin on an RNA-DNA Hybrid. *Biochem. biophys. Res. Commun.*, **65**, 815.

Halnan, K. E., Bleehen, N. M., Brewin, T., Deeley, T., Harrison, D., Howland, C., Kunkler, P., Ritchie, G., Wiltshow, E. & Todd, I. (1972) Early Clinical Experience with Bleomycin in the U.K. in Series of 105 Patients. *Br. med. J.*, iv, 635.

Ichikawa, T., Umesawa, H., Ohashi, S., Takeuchi, T., Ishizuka, M. & Hori, S. (1970) Animal Experiments Confirming the Specific Effect of Bleomycin against Squamous Cell Carcinoma. *Proc. 6th Int. Cong. Chemotherapy*, Tokyo, **2**, 315.

Ishizuka, M., Kakayama, H., Takeuchi, T. & Umezawa, H. (1967) Activity and Toxicity of Bleomycin. *J. Antibiot., Tokyo*, (A), **20**, 15.

Jorgensen, S. J. (1972) Time-dose Relationships in Combined Bleomycin Treatment and Radiotherapy. *Eur. J. Cancer*, **8**, 531.

Kunimoto, T., Hori, M. & Umezawa, H. (1967) Modes of Action of Phleomycin, Bleomycin and Forimycin on HeLa $S_3$ Cells in Synchronized Cultures. *J. Antibiot., Tokyo*, (A), **20**, 277.

Kuo, M. T. & Haidle, C. W. (1973) Characterization of Chain Breakage in DNA Induced by Bleomycin. *Biochim. biophys. Acta*, **335**, 109.

Kwok, C. S. & Chapman, I. V. (1977) The Effect of X-irradiation on Thymidine Transport Kinetics and DNA Synthesis in EAT Cells in Relation to Extracellular Thymidine Concentrations. *Int. J. Radiat. Biol.* (in press).

Matsuzawa, T., Onozawa, M., Morita, K. & Kakehi, M. (1972) Radiosensitization of Bleomycin on Lethal Effect of Mouse Cancer Cell *In vitro*. *Strahlentherapie*, **144**, 614.

Miyaki, M. & Ono, T. (1971) Inhibition of Ligase Reaction by Bleomycin. *J. Antibiot., Tokyo*, (A), **24**, 587.

Punnonen, R., Rantanen, J. & Gronroos, M. (1974) Effects of Bleomycin on $^3$H-TdR Incorporation in the Skin and on DNA, RNA and ATP Synthesis in the Primary Tumour of Patients with Vulval Carcinoma. *Ann. Chir. Gynaecol. Fenn.*, **63**, 146.

Suzuki, H., Nagai, K., Yamaki, H., Tanaka, N. & Umezawa, H. (1968) Mechanism of Action of Bleomycin. Studies with Growing Cultures of Bacterial and Tumour Cells. *J. Antibiot., Tokyo*, **21**, 379.

Suzuki, H., Nagai, K. & Yamaki, H. (1969) On the Mechanism of Action of Bleomycin: Scission of DNA Strands *In vitro* and *In vivo*. *J. Antibiot., Tokyo*, **22**, 446.

Terasima, T., Yasukawa, M. & Umezawa, H. (1970) Breaks and Rejoining of DNA in Cultured Mammalian Cells Treated with Bleomycin. *Gann*, **61**, 513.

Umezawa, H., Maeda, K., Takeuchi, T. & Okami, Y. (1966) New Antibiotics, Bleomycin A and B. *J. Antibiot., Tokyo*, (A), **19**, 200.

Umezawa, H., Takeuchi, T., Hori, S., Sawa, T., Ishizuka, M., Ichikawa, T. & Komai, T. (1972) Studies on the Mechanism of Antitumour Effect of Bleomycin on Squamous Cell Carcinoma. *J. Antibiot., Tokyo*, (A), **25**, 409.

Wharam, M. D., Phillips, T. L., Kane, L. & Utley, J. F. (1973) Response of a Murine Solid Tumour to *In vivo* Combined Chemotherapy and Irradiation. *Radiology*, **109**, 451.